# Genetic trends and trade-offs between growth and reproductive traits in a Nellore herd

**DOI:** 10.1371/journal.pone.0201392

**Published:** 2018-08-16

**Authors:** Luiza Rodrigues Alves Abreu, Virgínia Mara Pereira Ribeiro, Gabriela Canabrava Gouveia, Eduardo Penteado Cardoso, Fabio Luiz Buranelo Toral

**Affiliations:** 1 Departamento de Zootecnia, Escola de Veterinária, Universidade Federal de Minas Gerais, Belo Horizonte, MG, Brazil; 2 Fazenda Mundo Novo, Uberaba, MG, Brazil; University of Illinois, UNITED STATES

## Abstract

The knowledge of genetic trends and trade-offs between growth and reproductive traits might be useful to understand the evolution of these traits in livestock and natural populations of animals. We estimated the genetic trends and trade-offs between pre-weaning growth and calving intervals of Nellore animals from a commercial farm. Two-trait animal models were used to estimate covariance components and breeding values (EBV) for direct and maternal genetic effects of pre-weaning growth and direct genetic effects of calving intervals. Regression analyses were performed to examine the relationship between direct and maternal EBV of pre-weaning growth and direct EBV of calving intervals (dependent variables) and the coefficient of generation of each animal (independent variable). We also performed regression analyses to examine the relationship between direct EBV of calving intervals (dependent variables) and direct and maternal EBV of pre-weaning growth (independent variables). The genetic trends for direct and maternal genetic effect for pre-weaning growth were significant and presented genetic evolution in the studied Nellore herd. The genetic trends for the reproductive traits were also significant but indicated genetic changes in an unfavorable way. The genetic correlations between direct effects of pre-weaning growth and calving intervals traits and the genetic correlations between maternal effects of pre-weaning growth traits and direct effects of calving interval traits were not different from zero. The presence of trade-offs between the direct effects of growth and reproductive traits were confirmed through regression from direct EBV of calving intervals over EBV of pre-weaning growth traits. In addition, regression analyses showed that selection to increase pre-weaning growth also increased calving intervals. Our results showed that pre-weaning growth and calving intervals are increasing over generations and that trade-offs occurred between those traits in the studied Nellore herd.

## Introduction

Animal growth is important in livestock production and in natural populations because it is related with economic profit and adaptation [1–3]. In addition, artificial and natural selections have been effective in changing growth along generations [4,5]. In mammalian species, direct and maternal genetic effects affect growth. The direct genetic effects is the sum of the additive effects of each gene affecting a certain trait [6,7]. Maternal effect is defined as the dam’s phenotype contribution on the offspring’s phenotype [1]. It is possible to measure the maternal genetic effect by using the animal model that includes this effect. The maternal effect evaluation is mainly performed by pre-weaning growth traits because this is the moment that the calves are more dependent of the cows for feeding and protection. It is related to milk production and to parental investment that cows do in order to improve offspring performance [8,9], and it has genetic and environmental components [1].

Indirect selection for improving maternal effects occurs through selection for pre-weaning growth of the offspring because there is an association between the maternal genotype and the offspring’s phenotype [1,3,8]. However, the selection for the maternal effect can unfavorably affect the reproductive capacity of females [9], characterizing trade-offs between maternal effect and reproductive traits.

Trade-off between traits is the result of an unfavorable correlation between them [10]. The trade-offs between maternal effects and reproductive traits of parents are conflicts present in all life-history evolution [11]. This kind of trade-off may happen because of limitation of the available amount of energy to perform metabolic functions, such as the maintenance of the reproductive cycle [10,12,13]. It may also happen because of the differences between the genetic potential for milk production and reproductive physiology [12,13].

Trade-offs can occur at genetic or phenotypic levels and generally happen between different traits measured in the same individual [9,14] or in different individuals [15]. There are few studies that aimed at quantifying genetic trade-offs between maternal effect and reproductive performance of parents and, although evidences of their existence [9], significant effects were not found yet, because of lack of statistical power [14]. In this sense, studies that properly identify trade-offs at genetic level are important to understand the genetic basis that influence parental care and its influence in expressing the phenotype of interest in the populations. Therefore, our objectives were to evaluate the genetic trends and trade-offs between pre-weaning growth and reproductive traits in cattle (*Bos taurus indicus*). Genetic parameters and trends of pre-weaning growth and calving intervals were estimated. The trade-offs between direct and maternal genetic of pre-weaning growth and direct effects of calving intervals were inferred through genetic correlation and regressions analysis.

## Material and methods

### Data

Nellore is a zebu breed (*Bos taurus indicus*) developed in tropical regions and it accounts for approximately 40% of Brazilian cattle herd. The database used in this study contained records of Nellore animals born between 1994 and 2015 in a commercial herd. This herd underwent to selection for over 38 years and has animal records up to nine generations. The main selection criteria were related to growth traits. Data structure of this herd was suitable for this study because contained data regarding pre-weaning growth and reproductive traits of cows and regarding their calves for estimation of maternal genetic effects (Table A in S1 File). Animals were raised in a commercial farm located in Brotas, São Paulo state, Brazil (22°10’44.69”S and 48°01’20.9”W, 647 m of altitude, and Cfa Köppen-Geiger climate classification), until 2000. In 2001, the animals were transferred to another farm in Uberaba, Minas Gerais state (19°24’33.3”S and 48°06’34.5”W, 840 m of altitude, and Aw Köppen-Geiger climate classification). Animals were kept on pastures with free access to mineral supplementation throughout the year. The predominant grass (>80%) in both locations was from *Urochloa* genus and the stocking rate was approximately 0.98 animal units per hectare (one animal unit is equivalent to a 450-kg animal.). During the cow-calf phase, calves were kept with their dams in 30-hectare pastures and weaned at approximately 205 days of age.

When editing the database for body weight (BW), to calculate it in standard ages (in the case of BW120), information of animals with measurements performed between 75 and 165 days of age were utilized. From this body weight, birth weight was subtracted and the average daily gain was calculated and then the value obtained was multiplied by 120 days. Summing the value with birth weight, the body weight at 120 days of age (BW120) was obtained. Regarding BW205, the same procedure was realized, but with phenotypic data of animals between 160 and 250 days of age. These both measurements were considered as pre-weaning growth traits. Calving interval (CI),calculated as the difference between calving dates from two consecutive calvings, was used as reproductive trait considering the first, second, third, and fourth calving intervals (CI1, CI2, CI3, and CI4, respectively) when editing the data. We considered birth and calving date observations from 1994 to 2014. Calving intervals outside the range between 280 and 1448 days were discarded. Descriptive statistics regarding the data set used in the analyses are presented in Table 1.

**Table 1:** Summary statistics^1^ for pre-weaning growth and reproductive traits^2^ of Nellore cattle.

Trait	n	$\bar{X}$	sd	CV(%)	Minimum	Maximum
BW120, kg	16,062	123.99	18.62	15.02	41.81	197.83
BW205, kg	16,812	177.53	27.57	15.53	60.48	289.30
CI1, days	2,536	553.24	181.04	32.73	300.00	1,448.00
CI2, days	1,915	485.31	153.26	31.58	313.00	1,419.00
CI3, days	1,436	455.54	144.91	31.81	308.00	1,211.00
CI4, days	1,114	445.32	137.17	30.80	310.00	1,094.00

^1^n = number of records; $\bar{X}$ = mean; sd = standard deviation; CV = coefficient of variation

^2^ BW120 = body weight adjusted to 120 days of age; BW205 = body weight adjusted to 205 days of age; CI1 = first calving interval; CI2 = second calving interval; CI3 = third calving interval; CI4 = fourth calving interval.

### Models

The genetic analysis of maternal ability and reproductive traits was performed through two-trait animal model [16,17]. These animal models were used to obtain the genetic parameters and the estimated breeding values (EBV) in order to calculate the genetic trends and to infer about the presence of trade-offs. The bivariate animal model analyses were performed between pre-weaning growth and reproductive traits.

The general statistical model for pre-weaning growth was:

$$y_{ijklm}=\mu +a_{i}+m_{j}+pm_{j}+FE_{k}+b_{1}AGE_{l}+c_{1}ADC_{m}+c_{2}ADC_{m}^{2}+e_{ijklm},$$

in which *y*_*ijklm*_ represents the BW120 or BW205 for animal *i*; *μ* is the general constant present in all observations; *a*_*i*_ is the additive genetic effect of the animal *i*; *m*_*j*_ is the maternal genetic effect of the dam *j*, which is the animal’s mother; *pm*_*j*_ represents the permanent maternal environmental effect of the dam *j*; *FE*_*k*_ is the fixed effect of the *k* management group of the animal on the weighting day; *b*_1_ is the regression coefficient associated with the linear effect of the calf age to the evaluation (*AGE_l_*); *c*_1_ is the regression coefficient associated to the linear effect of the age of the dam at calving (*ADC*_*m*_); *c*_2_ is the regression coefficient associated to the quadratic effect of the age of the dam at calving ($ADC_{m}^{2}$); and *e*_*ijklm*_ is the associated error for each observation. The management groups were composed of animals from the same sex and raised in the same pasture. Groups with at least four animals were considered. The analyzed database had 882 and 954 groups for BW120 and BW205, respectively.

The general statistical model utilized for reproductive traits was:

$$y_{ijk}=\mu +a_{i}+EB_{j}+EC_{k}+e_{ijk},$$

in which *y*_*ijk*_ represents the CI1, CI2, CI3 or CI4 for animal *i*; *μ* is the general constant present in all observations; *a*_*i*_ is the additive genetic effect of the animal *i*; *EB*_*j*_ is the fixed effect group at birth *j*; *EC*_*k*_, is the fixed effect group at calving *k*; *e*_*ijk*_ is the associated error at each observation. The fixed effect groups at birth was composed of the following factors: year of birth, month of birth, and their interaction, totaling 90, 75, 65 and 60 groups for CI1, CI2, CI3 and CI4, respectively. The fixed effect group at calving was composed of calving year preceding to the interval, calving month, and their interaction, totaling 91, 86, 70 and 64 groups for CI1, CI2, CI3 and CI4, respectively. Detailed information about the statistical models and their assumptions can be found in the Supplementary Materials and methods.

### Genetic trends

Genetic trends of direct effects for pre-weaning growth and reproductive traits and maternal effects for BW120 and BW205 were calculated through linear regression of the EBV as a function of the generation coefficient of the animals. The generation coefficients were calculated according to the formula:

$$GCi=\left(\frac{GCS_{i}+GCD_{i}}{2}\right)+1$$

in which *GCi* represents the generation coefficient of the individual *i*; *GCS*_*i*_ is the generation coefficient of the sire of animal *i*; and *GCD*_*i*_ is the generation coefficient of the dam of animal *i*. Regression coefficients of the genetic trends were tested by using the F test.

### Regressions of estimated breeding value

The EBV for direct effects for reproductive traits (CI1, CI2, CI3 and CI4) were regressed as a function of maternal and direct EBV for pre-weaning growth traits (BW120 or BW205). These regressions were performed to base the inferences about the trade-offs. The regressions of direct EBV for reproductive traits in terms of direct EBV of BW120 or BW205 have considered information of cows having reproductive trait data and their own BW information (BW120 or BW205). In the regressions of the direct EBV of calving interval over maternal EBV of BW120 or BW205, we considered information of dams that had reproductive trait data and offspring with BW record (BW120 or BW205). In this way, all regressions have been considered the genetic values from animals that had phenotypic information for both traits.

## Results

### Genetic trends of pre-weaning growth and reproductive traits

Posterior means of heritabilities and correlations are presented together with their lower and upper limits of the highest posterior density intervals (HPD) with 90% of the samples (between parentheses). The direct heritabilities of BW120 and BW205 were 0.23 (0.17; 0.27) and 0.23 (0.18; 0.27), respectively. The maternal heritabilities of BW120 and BW205 were 0.10 (0.07; 0.13) and 0.08 (0.05; 0.11), respectively. The genetic correlations between direct and maternal effects of BW120 and BW205 were not different of zero and the posteriori means were 0.18 (-0.36; 0.00) and 0.02 (-0.19; 0.21), respectively. The ratio between the maternal permanent environmental effect and the phenotypic variances of BW120 was 0.14 (0.11; 0.16) and of BW205 was 0.14 (0.12; 0.16).

The direct heritabilities of first, second, third and fourth calving intervals were 0.09 (0.03; 0.14), 0.09 (0.04; 0.15), 0.10 (0.03; 0.17) and 0.10 (0.01; 0.20), respectively. Complementary information about covariances of pre-weaning growth and calving intervals can be found in Table B in S1 File.

Direct genetic trends of BW120 and BW205 were positive and significant (*P*< 0.0001), with average changes of 1.58 kg/generation for BW120 and 2.65 kg/generation for BW205 (Table 2 and S1 Fig). Maternal genetic trends of BW120 and BW205 were positive and significant (*P*< 0.0001), with average changes of 0.10 kg/generation for BW120 and 0.16 kg/generation for BW205 (Table 2 and S2 Fig).

**Table 2:** Estimates for genetic trends (standard error) of direct and maternal effects of pre-weaning growth traits^1^ on each two-trait analysis with calving intervals.

Trait^2^	Direct effect (kg/generation)		Maternal effect (kg/generation)	
	BW120	BW205	BW120	BW205
CI1	1.58 (0.03)	2.64 (0.04)	0.09 (0.01)	0.18 (0.02)
CI2	1.60 (0.03)	2.65 (0.04)	0.09 (0.01)	0.13 (0.01)
CI3	1.58 (0.03)	2.66 (0.04)	0.11 (0.01)	0.16 (0.02)
CI4	1.56 (0.03)	2.64 (0.04)	0.12 (0.01)	0.16 (0.02)

^1^BW120 = body weight adjusted to 120 days of age; BW205 = body weight adjusted to 205 days of age

^2^CI1 = first calving interval; CI2 = second calving interval; CI3 = third calving interval; CI4 = fourth calving interval; Each linear regression coefficient of the estimated breeding value was calculated according to each two-trait combination analysis; all linear regression coefficient were significant (*P*< 0.0001).

Direct genetic trends of calving intervals were also positive and different of zero (*P*< 0.0001), with average changes of 4.27, 3.20, 6.09 and 3.48 days/generation, for CI1, CI2, CI3 and CI4, respectively (Table 3 and S3 Fig).

**Table 3:** Estimates of genetic trends (standard error) of direct effects of calving intervals^1^ on each two-trait analysis with pre-weaning growth.

Trait^2^	Direct effect (days/generation)			
	CI1	CI2	CI3	CI4
BW120	4.23 (0.23)	3.00 (0.46)	5.00 (0.55)	2.60 (0.45)
BW205	4.31 (0.42)	3.39 (0.46)	7.18 (0.55)	4.36 (0.76)

^1^CI1 = first calving interval; CI2 = second calving interval; CI3 = third calving interval; CI4 = fourth calving interval

^2^BW120 = body weight adjusted to 120 days of age; BW205 = body weight adjusted to 205 days of age; Each linear regression coefficient of the estimated breeding value was calculated according to each two-trait combination analysis; all linear regression coefficient were significant (*P*< 0.0001).

### Trade-offs between direct effects of pre-weaning growth and calving intervals

**Fig 1:**
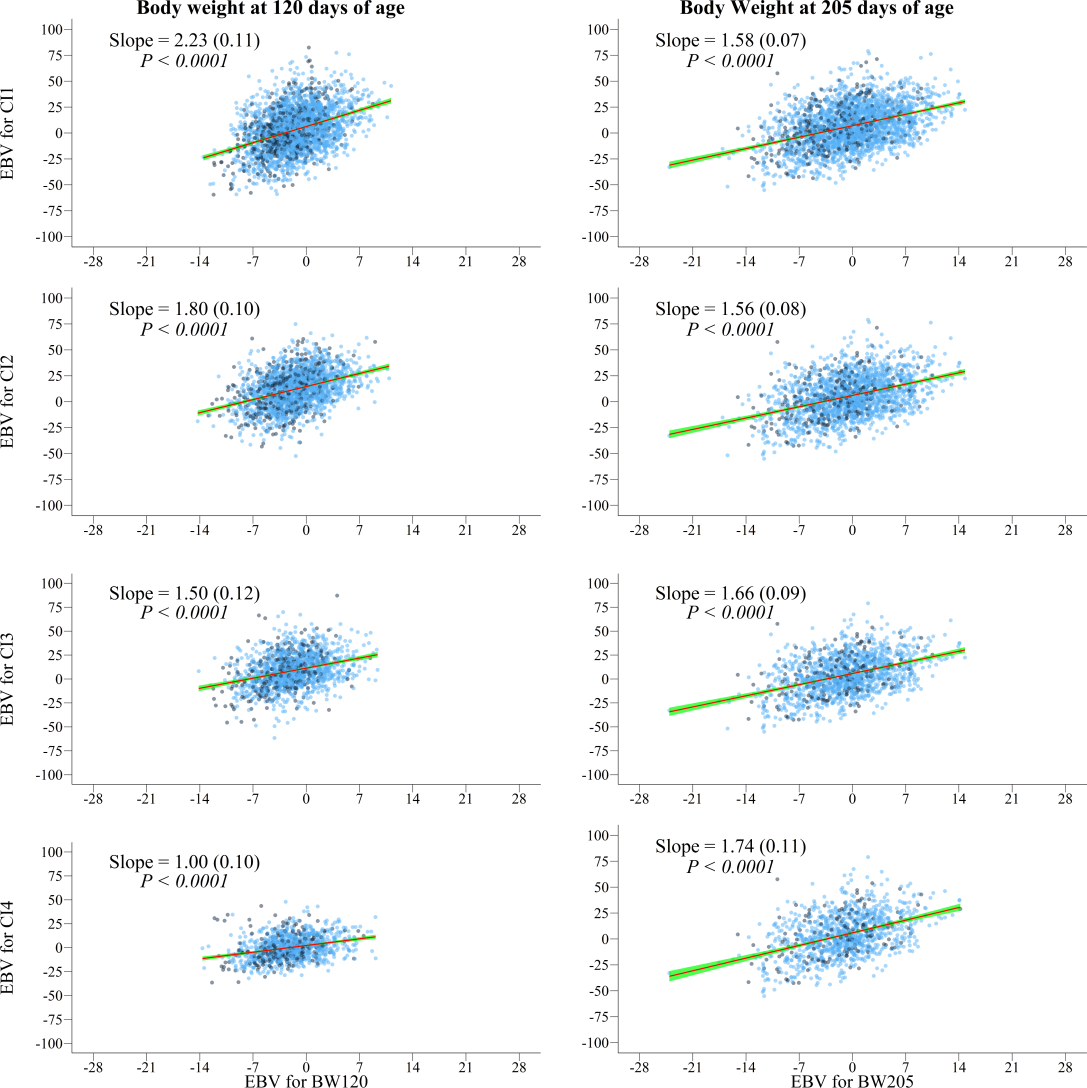
Variation of estimated breeding values (EBV) for direct effects of the first (CI1), second (CI2), third (CI3) and fourth (CI4) calving intervals over the EBV for direct effects of body weight at 120 and 205 days of age. The EBV for each animal is represented by the blue dots, the linear regression of EBV is represented by the red line) and the confidence interval is represented by the blue line). Slope (standard error) and significant level are depicted for each analysis of pre-weaning growth and calving intervals.

Genetic correlations between direct effects of pre-weaning growth (BW120 and BW205) and the direct effects of calving intervals did not differ from zero, except the genetic correlation between BW205 and CI3 (Table 4). However, the regression coefficients for direct EBV of reproductive traits over direct EBV of pre-weaning growth traits were statistically significant (P <0.0001, Fig 1 and Table C in S1 File).

**Table 4:** Posterior means (lower and upper limits of the highest posterior density interval with 90% of samples) of the correlations^1^ between pre-weaning growth^2^ and calving intervals^3^.

		CI1	CI2	CI3	CI4
*r*_*a*_	BW120	0.20 (-0.12; 0.56)	0.25 (-0.08; 0.61)	0.11 (-0.28; 0.55)	-0.06 (-0.57; 0.64)
	BW205	0.25 (-0.13; 0.62)	0.31 (-0.01; 0.62)	0.47 (0.09; 0.87)	0.17 (-0.20; 0.58)
*r*_*am*_	BW120	0.31 (0.04; 0.58)	0.16 (-0.13; 0.46)	0.37 (0.10; 0.68)	0.52 (-0.05; 0.96)
	BW205	0.33 (0.00; 0.64)	0.21 (-0.09; 0.52)	0.32 (-0.01; 0.62)	0.26 (-0.12; 0.58)
*r*_*e*_	BW120	0.00 (-0.07; 0.07)	0.02 (-0.06; 0.10)	0.06 (-0.03; 0.16)	0.07 (-0.04; 0.18)
	BW205	0.00 (-0.07; 0.07)	0.04 (-0.03; 0.12)	-0.04 (-0.13; 0.04)	0.02 (-0.08; 0.11)
*r*_*p*_	BW120	0.06 (0.01; 0.11)	0.08 (0.02; 0.14)	0.11 (0.04; 0.18)	0.10 (0.02; 0.18)
	BW205	0.07 (0.02; 0.12)	0.10 (0.04; 0.16)	0.07 (0.01; 0.14)	0.08 (0.00; 0.16)

^1^*r*_*a*_ = genetics correlations between direct effects; *r*_*am*_ = genetics correlations between maternal and direct effects; *r*_*e*_ = environmental correlations; *r*_*p*_ = phenotypic correlations

^2^BW120 = body weight adjusted to 120 days of age; BW205 = body weight adjusted to 205 days of age

^3^CI1 = first calving interval; CI2 = second calving interval; CI3 = third calving interval; CI4 = fourth calving interval; in red is the amount of samples below zero, in percentage.

The results showed that for every additional change (1 kg) in direct EBV of pre-weaning growth traits we can expect the calving intervals to increase by an average of 1.6 days (Fig 1 and Table C in S1 File).Which can be interpreted as the average change in the EBV of calving intervals for every additional change in the direct EBV of BW120 or BW205.

### Trade-offs between maternal effects of pre-weaning growth and calving intervals

**Fig 2:**
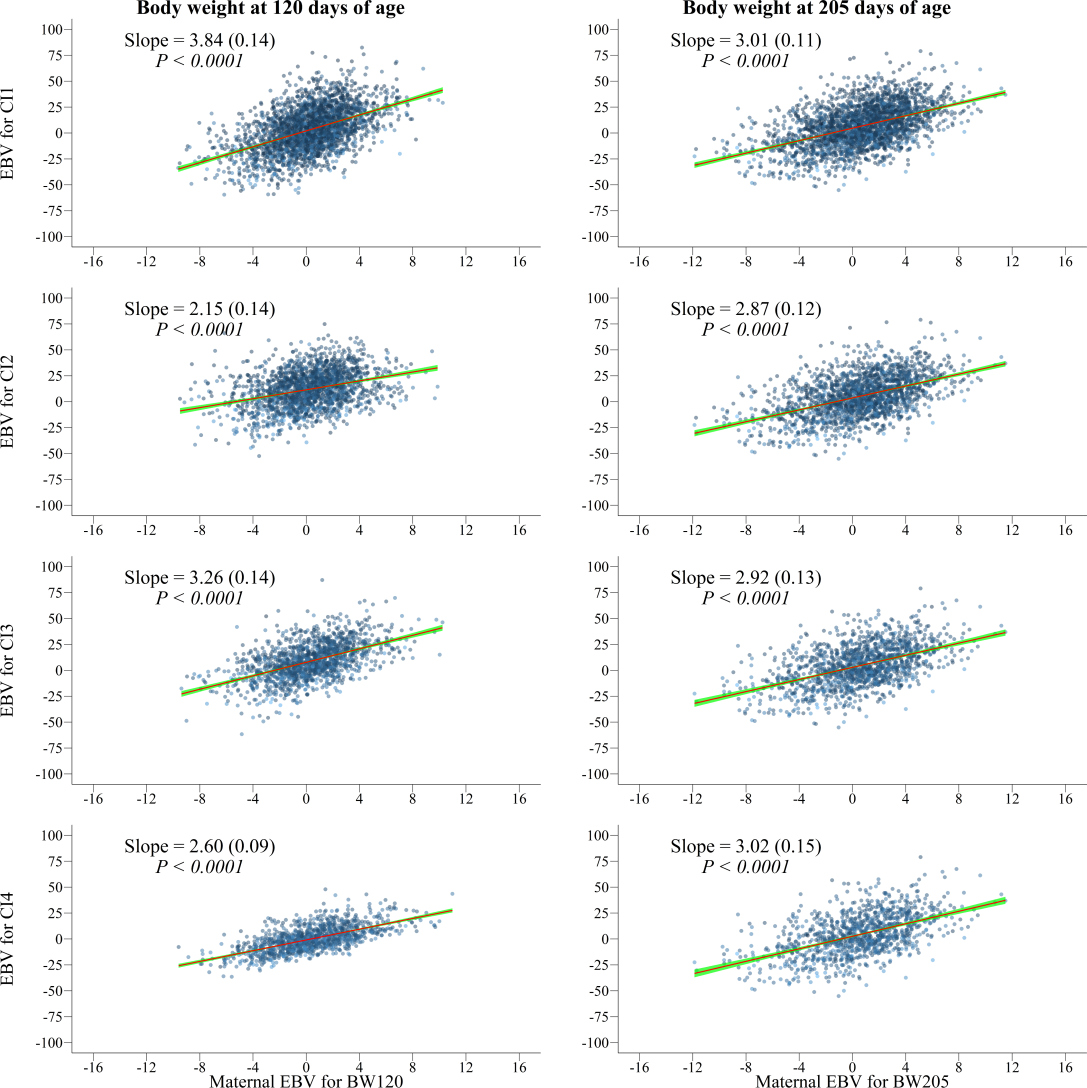
Variation of estimated breeding values (EBV) for direct effects for first (CI1), second (CI2), third (CI3) and fourth calving interval (CI4) over estimated breeding values (EBV) for maternal effects for body weight at 120 and 205 days of age. The EBV for each animal is represented by the blue dots, the linear regression of EBV is represented by the red line) and the confidence interval is represented by the blue line). Slope (standard error) and significant level are depicted for each analysis of pre-weaning growth and calving intervals.

In general, the genetic correlations between maternal effects of pre-weaning growth traits and the direct effect of calving intervals were not different from zero (Table 4). However, the regression coefficients for direct EBV of reproductive traits on maternal EBV of pre-weaning growth traits were statistically significant (*P* < 0.0001, Fig 2 and Table C in S1 File). One-kilogram increase in maternal EBV of pre-weaning growth traits will increase the calving interval by an average of 3 days (Fig 2 and Table C in S1 File).

At this point, it is also important to highlight that selection to increase genetic maternal effects of pre-weaning growth can be more impairing to dam reproduction compared with selection to increase genetic direct effects. To confirm this result we computed the confidence intervals for the regression coefficients for direct and maternal EBV and they were not overlapped (Table C in S1 File).

### Residual and phenotypic correlations between pre-weaning growth and calving intervals

Residual correlations between pre-weaning growth and calving intervals were not different from zero, and phenotypic correlations were of low magnitude but significant, except the phenotypic correlation between BW205 and CI4 (Table 4).

## Discussion

### Statistic model approach

The understanding of issues concerning trade-offs depends of the knowledge at both phenotypic and genetic levels [11]. In this sense, the statistical “animal” model allows to estimate genetic and non-genetic effects that affect multiple traits in animal populations along generations properly [16,17]. One previous study showed the existence of phenotype variance for trade-offs between offspring performance and reproductive traits [9], and another one failed to detect any additive genetic variances in offspring size and number probably because of insufficient statistical power [14]. Long term experiments (multiple generations), well designed data recording schemes and robust statistical model are needed to increase the accuracy and the precision of parameter estimates to infer about evolution, associations and trade-offs between traits.

In a long-term experiment, the modelling of year and season of birth, and management conditions effects are very important. In addition, when traits under evaluation are also affected by genetic and non-genetic maternal effects (e.g. BW120 and BW205), the statistical model needs to contemplate additional terms that control the factors which affect these traits.

Furthermore, the accuracy and the precision of covariance parameters estimates for direct and maternal effects are affected by the availability of phenotypic measurements of the dams and of their progenies [18]. And the accuracy and the precision of covariance parameters estimates in multiple trait analyses are also affected by the percentage of animals with phenotypic measurement of the multiple traits [19]. The multiple generations (nine generations) and phenotypic recording scheme (S1 File), and the robust animal model with genetic (direct and maternal), non-genetic (maternal permanent environment, age, contemporary groups, etc) effects we used in this study met these requirements and we expect our estimates are suitable for inference about our objectives, especially in mammalian species. The study was mainly based on the inference of the trade-offs between the pre weaning growth and reproductive traits.

### Genetic trends of pre-weaning growth and reproductive traits

The phenotypic records used in this study are from a commercial farm and selection has been performed to increase BW at weaning (≈ 205 days) and post-weaning (≈ 550 days). At weaning, the lighter calves (≈ 50% of males and 30% of females) are culled and the rest are kept for post-weaning evaluation. At the end of the post-weaning evaluation, only the young bulls with the highest pre-weaning growth rate are kept to replace old bulls and only the heifers that conceive before 30 months of age are kept to replace open cows.

Body weight has moderate heritability [20,21], and BW at different ages are genetically correlated [21,22]. Thus, improvement of BW120 and BW205 occurred as a consequence of indirect and direct selection [23] (*e*.*g*., selection for improving BW measured in further ages).

The direct genetic effects are larger than maternal genetic differences (Table B in S1 File) [20,24]. Maternal genetic effect depends of milk production, which has low to moderate heritability [24,25] and other maternal traits for example calf birth weight [25], that affect BW of calves indirectly. Thus, the phenotypic selection to increase BW will affect the averages of direct genetic effect but will not interfere on the averages of maternal genetic effect (Table 2, S1 and S2 Figs).

Our results showed that direct and maternal genetic effects of pre-weaning BW in cattle are not correlated. These are in disagreement with previous studies, which reported negative correlation (≈ -0.4) between direct and maternal effects [21,26]. However, statistical tests for correlations are not always reported and negative estimates might be more associated with data structure [18,27] than with the influence of genes that affect both direct and maternal effects [28]. The genetic correlation between the direct and maternal effect did not differ from zero and the genetic trend for each trait was positive, indicating an independent increase of the genetic potentials for the studied traits throughout generations (Table 2, S1 and S2 Figs).

Calving interval heritability is low [29,30] and unknown environmental factors and non-additive genetic effects are responsible for most of the differences between animals. Calving interval is not directly addressed in the selection program of the studied herd but cows that fail to calve in two consecutive seasons are culled. The positive genetic trends showed that this criterion is not effective to reduce calving intervals (Table 3 and S3 Fig) since unfavorable correlations between BW and CI can be observed. Thereby, the unfavorable increase in the average of calving intervals over generations might be indirectly caused by the selection of other traits, for instance, pre-weaning BW.

### Trade-offs between direct effects of pre-weaning growth and reproductive traits

The genetic correlations estimates of the present study (Table 4) and others from the literature [20,29,31] indicated lack of association between direct effect of pre-weaning growth and calving intervals in cattle. But the regression coefficients of direct effect of pre-weaning growth were significant indicating the existence of genetic trade-offs between direct effects of pre-weaning growth and reproductive traits (Fig 1 and Table C in S1 File). The breeding values regressed in function of the breeding value for the other trait allows to observe the genetic influence that each trait has over the other one. Regarding this regression it was possible to infer that animals with higher breeding values for the direct effect of the pre-weaning traits also have the highest breeding values for the direct effect of the reproductive traits. Thus, the effects possibly masked by the correlation analysis can be seen by regression analysis, which also verifies the relationship between two variables.

Body weights in different ages are moderately to highly correlated [21,32]. In this manner, selection to increase pre-weaning growth in early ages (pre- or post-weaning) will lead to an increase in BW at mature ages [22]. As cows became heavier along generations, their nutritional requirements also increase and energy supply from pasture may not be enough to meet their requirements [33]. Therefore, a negative impact in reproduction might occur [10].

### Trade-offs between maternal effects of pre-weaning growth and reproductive traits

Genetic correlations between maternal effects of pre-weaning growth and direct effects of calving interval were not significant to indicate the presence of trade-offs (Table 4). Weak genetic associations (0.02 to 0.04) between weaning BW and first calving interval were also reported in previous studies with cattle [20,29]. However, the regression coefficients of maternal effect were significant indicating the presence of genetic trade-offs between maternal effects of pre-weaning growth and reproductive traits (Fig 2 and Table C in S1 File).

Calves that are heavier at weaning, for instance, are more desirable in a livestock framework because they can reach the slaughter weight earlier than lighter calves. Notwithstanding, the cow needs to invest energy resources to wean a heavy calf. Increasing milk production is an expensive investment in this process, with an increase in energy requirement [34] and a negative impact in the reproduction endocrinology [12,13]. In addition, our results showed that selection for increasing pre-weaning growth will lead to an undesirable increasing in the calving intervals. In the artificial selection point of view, selection index or independent culling levels are useful selection methods to improve pre-weaning growth and reproductive traits in the desirable directions. In the natural selection point of view, those two groups of traits will evolve toward a balance. This increase in pre-weaning growth reflects on calving interval (Fig 2 and Table C in S1 File) and leads to small offspring number along the reproductive life [9].

### Residual and phenotypic correlations between pre-weaning growth and calving intervals

The residual correlations between pre-weaning growth and calving intervals were not significant (Table 4) and indicate that those environment factors that affect pre-weaning growth cannot be considered the same that affect calving interval. Pre-weaning growth and calving intervals are measured in different ages. While growth traits are assessed at 120 and 205 days of age, calving interval is assessed in 3-year-old animals, approximately, since this is the average age for the first calving in the evaluated herd. However, the residual correlation between pre-weaning growth and reproductive traits might be significant when both traits are measured in the same animal and at the same time [20].

The phenotypic correlations between pre-weaning growth and calving intervals were low but significant (Table 4). These values corroborate the estimate of 0.06 previously published by Mercadante et al. [20]. Phenotypic correlation is a combination of genetic and residual variances and covariances [35]. Despite the non-significant genetic and residual correlations, phenotypic correlations were associated with narrow highest posterior density intervals and were significant. The results presented in Table 4 showed that genetic correlations were not significant but they were the main causes of phenotypic correlations.

### Impact of selection for direct or maternal effects of pre-weaning growth traits on reproductive traits

We observed that regression coefficients of maternal effects were greater than regression coefficients of direct effects of pre-weaning growth traits (Figs 1 and 2, and Table C in S1 File). This result shows that the same increase in both maternal and direct effects of pre-weaning growth (one kilogram) will lead to different impairment in direct effect of calving intervals (~ 3 days vs ~ 1.6 days).

Maternal effect of pre-weaning growth is associated with milk yield [31] (and milk consumption), and milk yield is associated with reproductive endocrinology [13]. The selection for milk yield increases blood concentrations of somatotropin and prolactin, stimulators of lactation, and decreases insulin, hormone that is antagonistic to lactation, these changes in hormone concentration promote higher milk yield but may be potentially detrimental to other physiological functions, such as reproduction [36]. So, reproductive endocrinology affects calving interval through the interaction between reproductive and productive hormones. On the other hand, direct effect of pre-weaning growth is associated with growth itself, and its effect on reproduction is indirectly because it affects the nutritional requirements of the animals.

Selection is efficient to increase the means of pre-weaning growth traits in cattle. There are trade-offs between direct and maternal genetic effects of pre-weaning growth and direct genetic effects of reproductive traits in cattle. Therefore, the increase of female investment to wean heavier offspring brings negative effects on its reproductive performance.

## Supporting information

S1 Data Database with information of relationship matrix, phenotype and fixed effect groups of the studied animals. (XLSX)

S1 File Supplementary material and methods, and tables A, B, and C.
(DOCX)

S1 Fig Variation of estimated breeding values (EBV) for direct effects for body weight at 120 (left) and 205 (right) days of age (BW120 and BW205) along generations. The EBV of each animal is represented by gray dots, the means of EBV for each generation, by green dots), genetic trends of EBV, by red line, and the confidence interval by blue line. Slope (standard error) and significant level are showed for each analysis of pre-weaning growth and calving intervals.
(TIF)

S2 Fig Variation of estimated breeding values (EBV) for maternal effects for body weight at 120 (left) and 205 (right) days of age (BW120 and BW205) along generation. The EBV of each animal is represented by gray dots, means of EBV for each generation, by green dots, genetic trends of EBV, by the red line, and the confidence interval, by blue line. Slope (standard error) and significant level are showed for each analysis of pre-weaning growth and calving intervals.
(TIF)

S3 Fig Variation of estimated breeding values (EBV) for direct effects for first calving interval (CI1), second calving interval (CI2), third calving interval (CI3) and fourth calving interval (CI4) along generation. The EBV of each animal is represented by gray dots, means of EBV for each generation, by green dots, genetic trends of EBV, by red line and the confidence interval, by blue line. Slope (standard error) and significant level are showed for each analysis of pre-weaning growth and calving intervals.
(TIF)
