# Supplementary material for: Genetic trends and trade-offs between growth and reproductive traits in a Nellore herd
Source: PLoS One. 2018 Aug 16;13(8):e0201392. doi: 10.1371/journal.pone.0201392 (PMC6095503; doi:10.1371/journal.pone.0201392)
Supplement: S1 File — Supplementary material and methods, and tables A, B, and C. (DOCX) [file pone.0201392.s002.docx]

# **S1 File.** Supplementary materials and methods

The analyses were accomplished according to models previously described in the methodology, through Bayesian methods by using Gibbs sampler in two-trait analyses. In matrix notation, the general model used in the analyses was:

in which represents the vector with trait observations ( for BW120 or BW205) or (for CI1, CI2, CI3 or CI4); is the incidence matrix of fixed effects of trait ; represents the vector with the solution for fixed effects; is incidence matrices for direct additive genetic random effects of the trait; is the incidence matrix of the maternal genetic random effects for BW120 or BW205; is the incidence matrix of permanent maternal environmental random effects for BW120 or BW205; , and are vectors with solutions of direct additive genetic random effects, maternal and maternal permanent environment referents effects for BW120 or BW205; is the vector of associated residuals at each observation; , for BW120 or BW205 and for CI1, CI2, CI3 or CI4.

The assumed assumptions for random effects were:

in which represents the relationship matrix (25,343 animals); is the direct additive genetic variance for trait ; is the maternal genetic variance for BW120 or BW205; is the variance of the permanent maternal environmental effect for BW120 or BW205; is the residual variance for the trait; is the covariance between the and effects; is the identity matrix which order has the same number of dams with evaluated calves for the 1 trait, and and are identity matrices which orders have the same number of observations of traits.

The assumed *a priori* distributions for the parameters of interest were uniform distribution for the fixed effects , with normal distributions assumed for the random effects (, and ); inverse Wishart distributions, which were assumed for covariance matrices ( and ) and inverse chi-square distribution () was assumed for the variance , in which is the genetic covariance matrix; is the genetic covariance matrix between the pre-weaning growth and reproductive traits (the pre-weaning growth traits had the maternal and direct genetic effects and the reproductive traits had only the direct genetic effect); and are hyperparameters of the the inverse Wishart distribution; and and are degrees of freedom of inverse Wishart distribution of genetic covariances; and are the hyperparameters of inverse chi-square distributions; is the residual covariance matrix; is the residual covariance matrix between the pre-weaning growth and reproductive traits. Information about the *a posteriori* complete conditional distributions can be found in Sorensen and Gianola [37].

The complete conditional distribution samples were obtained through Gibbs sampler by using the GIBBS1F90 software [38]. We considered 1,100,000 chains with initial burn in of 100,000 samples and samples of covariance component values at each 100 cycles. The chain size were defined by preliminary analyses, according to the Raftery and Lewis [39] method, available in the BOA Package [40] of the R software (The R Foundation for Statistical Computing, 2015). The chains convergence was evaluated according to the criterion proposed by Geweke [41], also available in the same software, and by visual inspection of the sample values at each interaction.

The *a posteriori* distribution samples of each two-trait analysis were grouped in such a way that there were genetic parameters of 40,000 samples of pre-weaning growth traits and 20,000 samples of the reproductive traits. The calculated genetic parameters were heritability from direct () and maternal () effects and the ratio of permanent maternal environmental variance and the phenotypic variance (), as follow:

; ; ;

in which represents the phenotypic variance for pre-weaning growth traits; and represents the phenotypic variance for reproductive traits

The average of the analyzed genetic parameters were obtained as well as the highest posterior density interval with 90% of the *a posteriori* samples (HPD90) aiming to verify the uncertainty on the prediction. The high density intervals are created for each parameter of the model from the cumulative function distribution of the samples as the shortest interval to which the difference in the estimated values for cumulative distribution of samples at the end of each interaction is a nominal probability, assuming a unimodal distribution [42]. The correlation in which HPD90 values include zero value did not differ from zero.

# **Supplementary tables**

**Table A.** Number of records of pre-weaning growth^1^ and calving intervals^2^ (diagonal) and number of animals with pre-weaning growth and reproductive traits (above diagonal)

|  | BW120 | BW205 | CI1 | CI2 | CI3 | CI4 |
| --- | --- | --- | --- | --- | --- | --- |
| BW120 | 16,062 | 14,936 | 2,027 | 1,533 | 1,150 | 878 |
| BW205 |  | 16,812 | 2,157 | 1,637 | 1,238 | 955 |
| CI1 |  |  | 2,536 | 1,895 | 1,420 | 1,099 |
| CI2 |  |  |  | 1,915 | 1,435 | 1,113 |
| CI3 |  |  |  |  | 1,436 | 1,113 |
| CI4 |  |  |  |  |  | 1,114 |

^1^BW120 = body weight adjusted to 120 days of age; BW205 = body weight adjusted to 205 days of age; ^2^CI1 = first calving interval; CI2 = second calving interval; CI3 = third calving interval; CI4 = fourth calving interval.

**Table B.** Posterior means (lower and upper limits of the highest posterior density interval with 90% of samples) of the (co)variance components^1^ of pre-weaning growth^2^ and calving intervals^3^

|  | **BW120** | **BW205** | **CI1** | **CI2** | **CI3** | **CI4** |
| --- | --- | --- | --- | --- | --- | --- |
| $\boldsymbol{\sigma}_{\boldsymbol{a}}^{\boldsymbol{2}}$ | 51.42 | 91.84 | 2,517 | 1,845 | 1,859 | 1,834 |
|  | (39.39; 62.75) | (71.86; 111.90) | (842; 4,186) | (745; 2,908) | (583; 3,158) | (165; 3,617) |
| $\boldsymbol{\sigma}_{\boldsymbol{m}}^{\boldsymbol{2}}$ | 23.34 | 33.59 |  |  |  |  |
|  | (16.12; 30.27) | (22.74; 44.25) |  |  |  |  |
| $\boldsymbol{\sigma}_{\boldsymbol{am}}$ | -6.33 | 0.82 |  |  |  |  |
|  | (-3.10; 0.59) | (-9.78; 12.01) |  |  |  |  |
| $\boldsymbol{\sigma}_{\boldsymbol{mp}}^{\boldsymbol{2}}$ | 30.78 | 56.78 |  |  |  |  |
|  | (25.47; 35.73) | (47.55; 66.01) |  |  |  |  |
| $\boldsymbol{\sigma}_{\boldsymbol{e}}^{\boldsymbol{2}}$ | 128.01 | 223.50 | 26,432 | 17,706 | 16,606 | 15,901 |
|  | (121.10; 134.70) | (211.80; 235.20) | (24,680; 28,410) | (16,350; 19,030) | (14,990; 18,140) | (13,670; 18,020) |
| $\boldsymbol{\sigma}_{\boldsymbol{P}}^{\boldsymbol{2}}$ | 227.22 | 406.54 | 28,949 | 19,552 | 18,465 | 17,736 |
|  | (221.20; 233.18) | (395.61; 417.48) | (27,508; 30,367) | (18,384; 20,637) | (17,206; 19,659) | (16,389; 19,136) |

^1^$\sigma_{a}^{2}$ = additive genetic variance; $\sigma_{m}^{2}$ = maternal genetic variance; $\sigma_{am}$ = additive and maternal genetic (co)variance; $\sigma_{mp}^{2}$ = maternal permanent genetic variance; $\sigma_{e}^{2}$ = environmental variance; $\sigma_{P}^{2}$ = phenotype variance; ^2^BW120 = body weight adjusted to 120 days of age; BW205 = body weight adjusted to 205 days of age; ^3^CI1 = first calving interval; CI2 = second calving interval; CI3 = third calving interval; CI4 = fourth calving interval.

**Table C.** Estimates of regression coefficient for genetic trend and lower and upper limits of confidence interval with 90%^1^ (CI90) probability of direct and maternal effects of pre-weaning growth traits^2^

| Traits^2^ | BW120 | |  | BW205 | |  |
| --- | --- | --- | --- | --- | --- | --- |
|  | Slope (sd) | CI90 |  | Slope (sd) | CI90 |  |
|  | Direct effect (kg/day) | | | | |  |
| CI1 | 2.23 (0.11) | 2.06; 2.41 |  | 1.58 (0.07) | 1.46; 1.69 |  |
| CI2 | 1.80 (0.10) | 1.64; 1.97 |  | 1.56 (0.08) | 1.43; 1.69 |  |
| CI3 | 1.50 (0.12) | 1.31; 1.70 |  | 1.66 (0.09) | 1.51; 1.81 |  |
| CI4 | 1.00 (0.10) | 0.84; 1.15 |  | 1.74 (0.11) | 1.56; 1.92 |  |
|  | Maternal effect (kg/day) | | | | |  |
| CI1 | 3.84 (0.14) | 3.61; 4.06 |  | 3.01 (0.11) | 2.83; 3.18 |  |
| CI2 | 2.15 (0.14) | 1.92; 2.38 |  | 2.87 (0.12) | 2.68; 3.07 |  |
| CI3 | 3.26 (0.14) | 3.02; 3.49 |  | 2.92 (0.13) | 2.70; 3.14 |  |
| CI4 | 2.60 (0.09) | 2.44; 2.75 |  | 3.02 (0.15) | 2.77; 3.27 |  |

^1^The confidence intervals constructed were according to the equation in which: represents the value of the F test; is the variance of the error; is the effect of the independent variable of the model; and is the number of observations. BW120 = body weight adjusted to 120 days of age; BW205 = body weight adjusted to 205 days of age; CI1 = first calving interval; CI2 = second calving interval; CI3 = third calving interval; CI4 = fourth calving interval; within parenthesis is the standard error. Each linear regression coefficient of the calculated estimated breeding value was according to each combination analysis two-trait; all linear regression coefficient were significant (*P* <0.0001).

# **Supplementary References**

37. Sorensen D, Gianola D. Likelihood, Bayesian and MCMC methods in quantittative genetics. Springer; 2010.

38. Misztal I, Tsuruta S, Lourenco D, Aguilar I, Legarra A, Vitezica Z, et al. Manual for BLUPF90 family of programs [Internet]. Athens: University of Georgia; 2015. Available: ftp://tech.obihiro.ac.jp/suzuki/blupf90_all2.pdf

39. Raftery AE, Lewis SM. One long run with diagnostics: implementation strategies for Markov chain Monte Carlo. Stat Sci. 1992;7: 492–497. doi:10.1214/ ss/1177011143

40. Smith BJ. Bayesian output analysis program (BOA) version 1.1 user’s manual [Internet]. 2005. Available: http://www.public-health.uiowa.edu/boa/boa.pdf

41. Geweke J. Evaluating the accuracy of sampling-based approaches to the calculation of posterior moments. Bayesian Stat 4. 1992; 169–193.

42. Plummer M, Best N, Cowles K, Vines K. Package “coda.” R News. 2016; Available: ftp://cran.wu-wien.ac.at/pub/R/web/packages/coda/coda.pdf
